# A311 EFFICACY OF PANCREATIC CANCER SCREENING IN HIGH RISK INDIVIDUALS

**DOI:** 10.1093/jcag/gwad061.311

**Published:** 2024-02-14

**Authors:** B Harjot, D Cheng, J Buttar, E Lam, J J Telford

**Affiliations:** Gastroenterology, The University of British Columbia Faculty of Medicine, Vancouver, BC, Canada; The University of Western Australia Faculty of Health and Medical Sciences, Perth, Western Australia, Australia; Gastroenterology, The University of British Columbia Faculty of Medicine, Vancouver, BC, Canada; Gastroenterology, The University of British Columbia Faculty of Medicine, Vancouver, BC, Canada; Gastroenterology, The University of British Columbia Faculty of Medicine, Vancouver, BC, Canada

## Abstract

**Background:**

Pancreatic cancer (PC) is the 11th most prevalent cancer in Canada with about 6900 new cases in 2021. Due to late diagnosis, it is the 4th largest cause of cancer related mortality in Canada. Despite advances in diagnosis and treatment over the past decade, the 5-year survival remains ampersand:003C10%, making pancreatic cancer one of the most lethal malignancies worldwide. However, if detected early, the 5-year survival increases to 80%. The incidence of pancreatic cancer remains low. Therefore, the International Cancer of the Pancreas Screening (CAPS) consortium do not recommend screening everyone. Selective screening in high-risk individuals may be effective in early detection and treatment of pancreatic cancer.

**Aims:**

1. To determine the characteristics of patients undergoing PC screening at a single tertiary care center in Vancouver.

2. To determine the frequency of disease progression and outcomes in patients diagnosed with high-risk lesions during screening.

**Methods:**

Retrospective chart review was performed for patients who were screened between January 1, 2005 to June 30, 2023. As per consensus from the CAPS Consortium, patients with at least 2 close relatives with PC, and those with predisposing genetic mutations screened using endoscopic ultrasound (EUS) and/or magnetic resonance imaging (MRI) were included in the study. Clinical data, such as age, tobacco use, genetic risk, endoscopic findings, and outcomes, such as disease progression, were collected. Statistical analysis was performed using Rstudio and Microsoft Excel.

**Results:**

73 patients were included, with 42 included due to pathogenic germline mutations, and 31 with familial pancreatic cancer. Most were female (52, 71.2%). Median age in the study was 54 years (range 24-74 years) at initial screening. 66 patients (90.4%) received an EUS and 7 patients (9.6%) received MRI for initial screening. At the time of initial screening. 74.6% of patients had a normal pancreas. 6 patients (8.2%) were found to have neoplastic lesions, e.g. ampulloma, neuroendocrine tumour or adenocarcinoma at initial screening and referred for surgery. In the median follow up time of 67.9 months (range 0-168.1 months), 6 patients were noted to have disease progression, 4 of which had increase in cyst size without any other worrisome features. 2 patients had progression to malignancy. 1 of those patients received chemotherapy and surgical resection and remains alive; the other patient was lost of follow up due to geographical move.

**Conclusions:**

Pancreatic cancer screening in high risk individuals is effective in early detection and treatment. Given lower incidence of pancreatic cancer, we likely need additional patient enrolment to observe a significant improvement in patient outcomes.

**Funding Agencies:**

None

